# Identification of blood biomarkers of a healthy dietary pattern as facilitated by cluster analysis in patients from the MEDDINI study: a pilot randomised trial

**DOI:** 10.1017/jns.2026.10115

**Published:** 2026-07-07

**Authors:** Shirin Macias, Ali Yilmaz, Joseph Kirma, Sarah E. Moore, Michelle C. McKinley, Pascal P. McKeown, Jayne V. Woodside, Stewart F. Graham, Brian D. Green

**Affiliations:** 1 Institute for Global Food Security, School of Biological Sciences, Queen’s University Belfast, Biological Sciences Building, Belfast, BT9 5DL UK; 2 Corewell Health Research Institute, Metabolomics Department, Royal Oak, MI 48073, USA; 3 Corewell Health East William Beaumont University Hospital, Royal Oak, MI 48073, USA; 4 Oakland University-William Beaumont School of Medicine, Rochester, MI 48309, USA; 5 Centre for Public Health School of Medicine, Dentistry and Biomedical Sciences, Queen’s University Belfast, Institute of Clinical Science, Belfast, BT12 6BJ, UK; 6 School of Medicine, Dentistry and Biomedical Sciences, Queen’s University Belfast, Belfast BT9 7BL, UK

**Keywords:** Betaine, Biomarkers, Citrate, Dietary patterns, Fatty acids, *K*-means, Metabolomics, Plasma, Serum

## Abstract

Dietary biomarkers may help objectively assessing dietary pattern adherence. This study performed *K*-means clustering analysis on quantitative food diary data from a dietary intervention study. Standardised dietary data (134 food diaries) from 57 participants were *K*-means clustered stepwise until fully optimised and cross-validated. The primary endpoint was to develop distinct dietary clusters and to evaluate the performanceof 90 plasma metabolites. The secondary endpoint was to analyse the biomarker-food groups relationships from those distinct dietary patterns. The final two cluster models comprised of 6 specific food types. Cluster 1 included participants with higher intake of fruit and vegetables, legumes, fish and whole grain cereals, and lower intake of meat and sweet foods than Cluster 2. Ten plasma metabolites significantly differed between the clusters (*p* < 0.05; q < 0.05) with reasonable biomarker performance (receiver operating characteristic (ROC): 0.64–0.72). Docosahexaenoic acid (DHA), eicosapentaenoic acid (EPA), α-linolenic acid, citric acid and vitamin C were significantly higher in Cluster 1, whereas adrenic acid, osbond acid, cholesterol, dihomo-γ-linolenic acid (DGLA) and triglycerides were higher in Cluster 2. Five additional metabolites also showed significant differences (*p* < 0.02; q < 0.11) and were included: palmitic acid, tyrosine, β-carotene, α-carotene and betaine. The DHA-to-Osbond acid ratio was an optimal indicator distinguishing healthy from unhealthy dietary patterns (ROC: 0.78). Combining clustering and metabolite profiling methods effectively identifies biomarkers of particular dietary patterns and highlights several robust food-metabolite correlations.

## Graphical abstract

**Figure:**
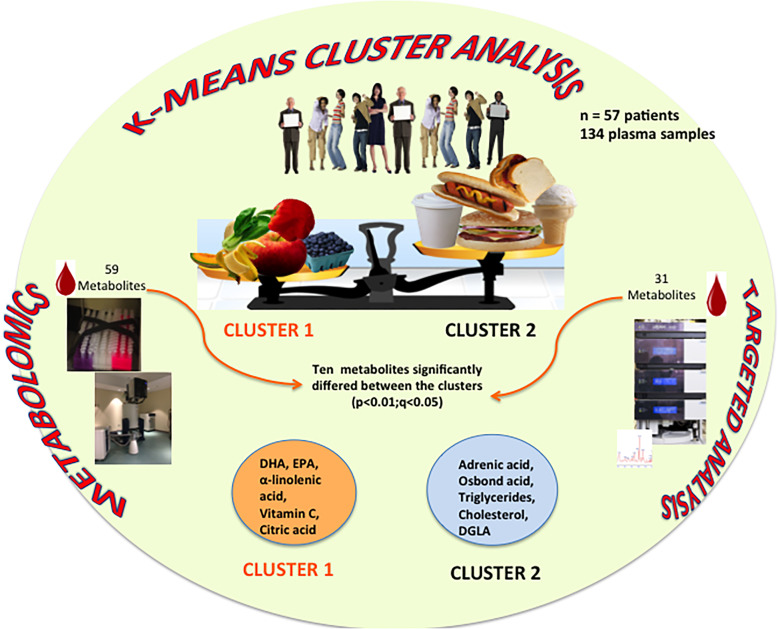

## Introduction

Biomarkers of particular dietary patterns could play a key role in monitoring dietary intake, aid in the determination of the health benefits of certain eating patterns, and potentially inform dietary guideline advice.^(1)^ Using newly identified biomarkers in combination with traditional targeted approaches could provide a more robust panel of biomarkers associated with dietary intake.^(2)^

Putative biomarkers are indicators tentatively linked to the consumption of specific foods or adherence to dietary patterns, offering valuable insights into dietary intake. Several food biomarkers have been identified, such as carnitine for red meat, caffeic acid for coffee, fatty acids for fish, isoflavones for soy products, resveratrol for wine, alkylresorcinols for whole-grain consumption, hippuric acid for fruit and vegetable intake, and proline betaine for citrus fruits.^(3)^ While these biomarkers reflect individual food consumption, the intake of single foods does not account for the totality of dietary impact on health.^(4)^ Dietary patterns, however, represent a broader picture of food and nutrient consumption, thus multiplexed biomarker panels assessing overall dietary intake would be of value.^(5)^ Short-chain fatty acids derived from dietary fibre fermentation have been proposed as markers of plant-based diets^(6)^ while carotenoids and flavonoids are potential biomarkers of fruit and vegetables and may be highly correlated to their intake.^(7)^ Certain metabolites have been associated with adherence to healthy dietary patterns, among them metabolites related to the metabolism of carbohydrates (3-hydroxybutyrate, citrate, and cis-aconitate), and lipid metabolism (oleic and suberic acid) were linked to the Mediterranean diet (MD)^(8)^ and metabolites linked to fish and whole grain intake such as Phosphatidylcholine (18:0/22:6) and pipecolic acid betaine respectively were reported as biomarkers of the New Nordic Diet.^(9)^ Yet, many of these biomarkers need to be individually validated in dose–response intervention studies before they can be put into practical use. An alternative approach is to identify typical dietary patterns, based on the intake of each food type, then to identify prominent biomarkers which best reflect that particular pattern and validate them.^(10)^

Cluster analysis is an a posteriori analytical approach used to identify dietary patterns and classifying individuals into distinctive, non-overlapping groups. Several studies have applied K-means cluster analysis to dietary intake data;^(11)^ Such an approach has demonstrated many advantages such as straightforward mathematical hypothesis, fast convergence, and easy implementation.^(12)^

Identifying dietary patterns using cluster analysis may reveal biologically meaningful biomarkers,^(11)^ although its use in this way has been quite limited.^(13)^ In an urban Chinese population, three dietary patterns were derived, and a pattern of high intake of refined cereals was associated with elevated homocysteine^(14)^ (linked to cardiovascular disease (CVD) risk)^(15)^ and reduced vitamin B12.^(14)^ Gibbons et al. used cluster analysis to develop a model for classification of people (*n* = 567) according to distinct dietary patterns using metabolomic data. Among the identified metabolites, betaine and citrate were significantly higher in cluster 1 and creatinine and N-acetylglutamate in cluster 2. Their model was successfully validated in an independent cohort which revealed a percentage of misclassification of only 6%.^(16)^

A priori-defined dietary patterns, e.g. healthy Nordic food index (HNFI) or the Baltic Sea diet score (BSDS)^(17)^ have been combined with metabolomics data to discover biomarkers of healthy eating. However, there have been few attempts to do this using a posteriori approach.^(13)^

The primary objective of the present investigation was two-fold: to perform K-means clustering analysis on quantitative food diary data from participants enrolled in the ‘Mediterranean diet in Northern Ireland’ (MEDDINI) intervention study to develop distinct dietary pattern clusters, and to identify blood biomarkers associated with such dietary patterns. For this, an extensive list of 90 potential plasma metabolites were evaluated for their ability to assign individuals to each of the derived dietary clusters. A secondary goal of this study was to analyse the biomarker-food groups relationships from those distinct dietary patterns.

## Materials and methods

### Study participants and dietary data collection

The MEDDINI study was an interventional pilot randomised controlled parallel group trial aiming to examine whether participants in a Northern European population would adopt and maintain a MD. Sixty-one willing participants from a Northern European population, were recruited (samples from *n* = 57 participant were available for the present study) from the Cardiology Directorate, Royal Victoria Hospital, Belfast. Because the MEDDINI intervention was a pilot study examining whether participants would comply with MD advice and there were no relevant data available for the population in Northern Ireland, power/sample size calculations were not considered to be appropriate. Participants recruited were aged between 39 and 78 years and had a recent diagnosis (within four weeks prior to starting the study) of myocardial infarction (MI) or unstable angina. Participants with severe heart failure, pending on immediate inpatient coronary revascularisation, on warfarin (Coumadin) therapy, on omacor (Eicosapentaenoic acid, docosahexaenoic acid) therapy, with cognitive impairment, those with records of extreme alcohol intake, taking multivitamin/fish oil supplements, unable to comply with the diet or to provide informed consent or those who were not expected to live longer than 6 months for any other causes were excluded from the study.

The MEDDINI intervention was a 12-month longitudinal study, and blood samples were collected at baseline, 6 months and 12 months. However, all participants made dietary changes towards a healthy dietary pattern throughout the intervention, thus, timepoints were not taken into consideration. Seven-day food diaries were used to collect food consumption data. Participants were asked to record the foods consumed over seven consecutive days, providing extensive detail, and including an estimation of quantity consumed and information on preparation methods used. From seven-day food diaries, a database was created registering all food amounts eaten by all participants during the intervention. All food amounts were registered in grams in the database. Completion of original study was carried out in 2006 and trial registration was not required at the time. This study was conducted according to the guidelines laid down in the Declaration of Helsinki and all procedures involving human subjects were approved by the Queen’s University Belfast Research Ethics Committee; Ethical approval references: RGHT000049 and 15.42 for the MEDDINI original study and for the latest analysis respectively. Written informed consent was obtained from all subjects.

Participants’ food diary data collected during the MEDDINI study were used in the present study to apply K-means cluster analysis. Available data from the 57 participants (i.e., gender, BMI, blood pressure, and smoking status) were collected and analysed. Independent t-tests were conducted to examine significant differences in age, BMI, and blood pressure.

### Sampling and analysis

Fasting blood samples were collected to identify and assess nutritional biomarkers. Targeted, biochemical variables were measured in the Nutrition and Metabolism Group, Centre for Clinical and Population Science (now Centre for Public Health), Queen’s University Belfast. Serum retinol, α- and γ-tocopherol, lutein, zeaxanthin, β-cryptoxanthin, lycopene, α- and β-carotene by high performance liquid chromatography with diode array detection.^(18)^ Plasma vitamin C was assessed by fluorometric assay using a Cobas FARA analyser (Roche Diagnostics, Basel, Switzerland).^(19)^

Serum folate and vitamin B12 were measured by radioassay using a commercially available kit (ICN Pharmaceuticals Ltd.). Serum lipids were measured (total cholesterol, high-density lipoproteins (HDL) cholesterol, and TG) by enzymatic assays (Randox Ltd., Crumlin, NI) on an I-Lab 600 autoanalyser, and plasma fatty acids measured by gas chromatography.^(20)^ All assays had intra- and inter-batch coefficients of variation that were within acceptable limits (<10%). A total of 59 metabolites were identified and analysed using one-dimensional proton nuclear magnetic resonance 1D 1H-NMR spectroscopy, the details of which have been described previously.^(21)^ Briefly, plasma samples were filtered using pre-washed (x7) 3.5 KDa filters (Amicon Micron YM-3; Sigma–Aldrich, St. Louis, MO) via centrifugation at 13,000 g, at 4°C for 30 min to remove macromolecules, then deuterium oxide (D2O) and a buffer containing the internal standard disodium-2,2-dimethyl-2-silapentane-5-sulphonate (DSS) was added. Samples (200 μL) were subsequently transferred to a standard Bruker 3 mm NMR tube for analysis. Data collection was carried out on a 600 MHz Bruker ASCEND NMR spectrometer equipped with a 5 mm TCI cryoprobe using a randomised order with two hundred and fifty-six transients acquired. Chemical shifts were reported in parts per million (ppm) of the operating frequency. Bayesil web-based software was used to identify and quantify metabolites.^(22)^

### Statistical analysis

All data from the MEDDINI study, including 31 nutritional biomarkers and CVD risk factors, were analysed. Additionally, non-targeted ^1^H-NMR spectroscopic profiling identified and quantified 59 metabolites, representing all recoverable metabolites from the spectra, yielding a dataset of 90 metabolites.

Distinct dietary patterns were derived using K-means cluster analysis in SPSS (IBM SPSS Statistics 26). Food intake data was transformed into standardised *z*-scores and the maximum number of iterations was 10. This number was determined by the default settings in SPSS, which are based on tested protocols to ensure a balance between computational efficiency and convergence.^(23)^ Clustering was conducted in a stepwise manner sequentially altering the number of clusters until fully optimised and cross-validated. Dietary data collected from food diaries included the following groups: (‘fruit, fruit juice and vegetables’, ‘legumes’, ‘fish’, ‘red, white and processed meat’, ‘whole grain cereals’, ‘sweet foods’, ‘nuts’, ‘alcohol’ and ‘olive/rapeseed oil’). Food types were only removed from the clustering model if their intake was rare, atypical, or consumed by a very small number of participants. Only 3 food groups (nuts, alcohol and olive/rapeseed oil) were excluded from clustering analysis because their consumption was rare, consumed by a very small number of participants, or its data was highly skewed. Building in the methodology of previous research^(24,25)^ a rigorous 3-fold cross-validation procedure was performed, where samples were randomly divided into 3 equal parts and cluster analysis was re-run but with one-third of the samples held back. This procedure was repeated at random 10 times, and the model was deemed valid if a similar clustering pattern was achieved and if SPSS confirmed that convergence was achieved for that model on at least 8 out of 10 iterations. A methodical optimisation approach was undertaken where the number of clusters was varied between 2 and 4, and with the food groups included in models being varied between 4 and 7. The goal was to achieve a clustering approach which was consistent, resulted in balanced sample sizes, and was nutritionally meaningful.^(11)^ Differences in food group intakes between clusters were assessed using a t-test in SPSS. For visualisation of the 2-cluster model, a mean plot with 95% confidence intervals (CIs), and PCA plots for the 2-cluster and 3-cluster models (the latter for comparison) were generated in SPSS.

For biomarker analysis, metabolite data was first tested for normality (Shapiro–Wilk) in SPSS. Subsequent analyses were conducted in MetaboAnalyst (version 4.0).^(26)^ Comparisons between Cluster 1 and Cluster 2 were performed by t-test to obtain p-values which underwent a Bonferroni false discovery rate (FDR) correction (*q*-values) to account for multiple comparisons. Receiver operating characteristic (ROC) curve analysis was conducted to assess biomarker performance. Area under the ROC (AUROC) values were obtained for each metabolite but also for all possible pair-wise metabolite ratios. Auto-scaled metabolite data were visualised by Principal Component Analysis (PCA) and Partial Least Squares-Discriminant Analysis (PLS-DA). Variable importance in projection (VIP) plots from PLS-DA models were used to identify the most influential metabolites for separating groups. For the secondary outcome of this study, non-parametric food-metabolite correlations were performed (Spearman’s rank correlation coefficient) on SPSS and p-values underwent Benjamini–Hochberg correction (Q-values) to account for multiple comparisons and were deemed significant if *p* ≤ 0.05 and Q ≤ 0.05.

## Results

### K-means clustering analysis

A total of 134 plasma samples from 57 subjects underwent high-performance liquid chromatography (HPLC) and 1D 1H-NMR analysis and therefore only food diary data from these samples underwent K-means cluster analysis. Table S1.A showed the baseline characteristics of the MEDDINI study participants included in the present study (*n* = 57), and Table S1.B summarised the characteristics of participants in Cluster 1 and Cluster 2 (Supporting Information). Mean age was 57 for the Cluster 1 and 56.68 for the Cluster 2. Mean BMI was 30.25 and 30.07 respectively. No significant differences were observed in age, BMI, smoking status, sex distribution, and systolic blood pressure between clusters (*p* > 0.05). A statistically significant difference was observed in diastolic blood pressure between clusters (*p* = 0.014) (Supporting Information Table S1.B).

**Figure 1.**
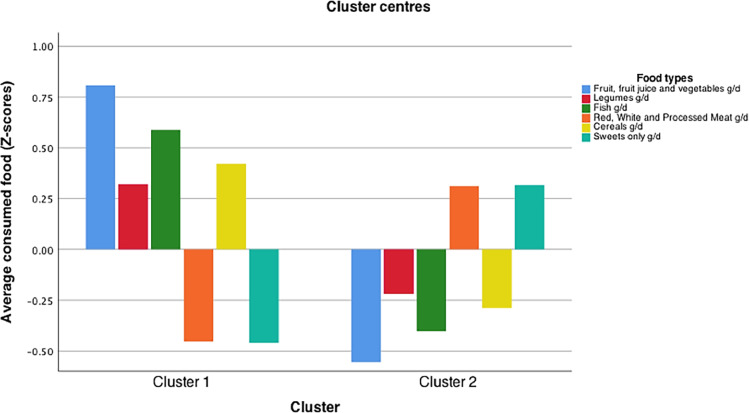
Figure 1 long description. The optimised and cross-validated K-means cluster model of dietary intake data from the MEDDINI study. Model developed using the K-means cluster algorithm in SPSS (version 26). Y-axis shows food intake data represented as standardised *z*-scores. X axis the resultant clusters after optimisation and cross validation. Food groups included into the cluster model were: ‘fruit, fruit juice and vegetables’, ‘legumes’, ‘fish’, ‘red, white and processed meat’, ‘whole grain cereals’, ‘sweet foods’.

Models with more than 2 clusters (k >2) were found not to be optimal. The maximum number of iterations in SPSS was reached without convergence in more than 8 out of 10 repeated runs. Models with fewer food groups (4 or 5) did not produce nutritionally meaningful clusters. Models with more than 6 food groups led to unbalanced sample sizes. Following cross-validation, a 2-cluster model was adopted, composed of 6 food types (‘fruit, fruit juice and vegetables’, ‘legumes’, ‘fish’, ‘red, white and processed meat’, ‘whole grain cereals’, ‘sweet foods’). These two optimal clusters were ‘Cluster 1’ comprised of 53 samples and ‘Cluster 2’, comprised of 81 samples. The different intake patterns emerging from each cluster are shown in Figure 1. Cluster 1 was characterised by higher consumption of fruit, fruit juice and vegetables, legumes, fish and whole grain cereals and a lower consumption of red, white and processed meat and sweets whereas Cluster 2 was characterised by a lower intake of fruit, fruit juice and vegetables, legumes, fish and whole grain cereals and a higher intake of red, white and processed meat and sweet foods.

**Figure 2.**
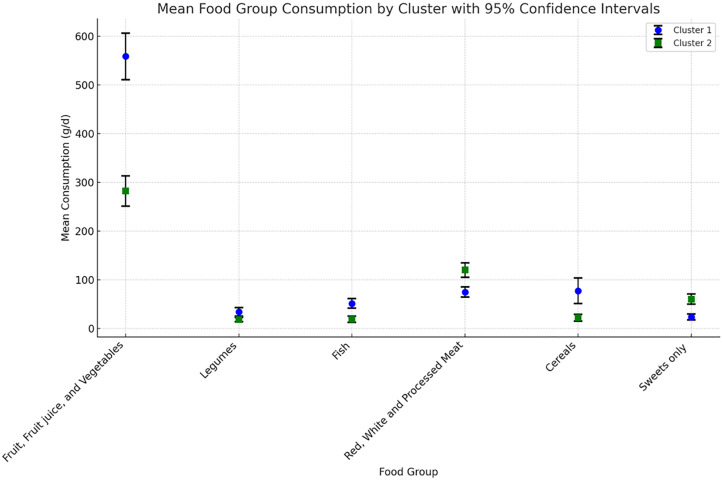
Figure 2 long description. Comparative analysis of dietary patterns in cluster 1 and cluster 2. The figure displays the average intake of 6 food groups (fruit, fruit juice, and vegetables; legumes; fish; red, white, and processed meat; cereals; and sweets) based on the 2-cluster solution identified using k-means clustering. Error bars represent 95% confidence intervals for the mean values. Non-overlapping intervals highlight distinct dietary patterns between clusters, supporting the validity of the 2-cluster model.

Average daily amounts consumed by individuals from each cluster and their respective 95% CIs are shown in Table 1 and illustrated in a mean plot (Figure 2). The t-test showed significant (*p* < 0.05) differences in all food variables between Clusters 1 and 2 (Table 1). PCA plot with centroids illustrated cluster separation and distinct dietary patterns for the 2-cluster model and the 3-cluster model. The latter did not show improved separation compared to the 2-cluster model (Supporting information Figure S1).

**Table 1.** Differing food intake recorded for the two dietary clusters developed by K-means clustering. Average and standard deviation (SD) for the 6 food types used to develop the K-means cluster model. P-values are from t-tests comparing food group amounts between cluster 1 and cluster 2. Differences between food groups were deemed statistically significant if *p* < 0.05 Table 1 long description.

Food type	Cluster 1 (*n* = 53) (g/day)	95% CI	Cluster 2 (*n* = 81) (g/day)	95% CI	*p*-value
Fruit, fruit juice and vegetables	569.44 ± 182.78	± 49.21	281.30 ± 137.22	± 29.88	7.12E–16
Legumes	32.48 ± 32.18	± 8.66	18.27 ± 19.55	± 4.26	1.9E–3
Fish	53.89 ± 35.01	± 9.43	18.41 ± 28.55	± 6.22	2.58E–07
Red, white and processed meat	74.72 ± 37.00	± 9.96	121.64 ± 67.34	± 14.66	2.27E–06
Wholegrain cereals	27.15 ± 18.15	± 4.89	16.70 ± 24.76	± 5.39	1.5E–4
Sweet foods	26.22 ± 23.69	± 6.38	58.85 ± 46.56	± 10.14	1.58E–08

### Univariate and multivariate analysis of metabolites

Metabolite data was found to be non-normally distributed following Normality (Shapiro-Wilk) testing, and thus non-parametric statistical tests were performed. Ten of the 90 metabolites measured in plasma significantly differed between the two clusters (*p* < 0.05; *q* < 0.05) (Table 2). Samples from Cluster 1 had significantly higher blood levels of DHA, EPA, α-linolenic acid, citric acid, and vitamin C. Compared with Cluster 2 these metabolites were 22%, 25%, 20%, 17% and 22% higher, respectively. Individuals from Cluster 2 had higher levels of adrenic acid, osbond acid, cholesterol, DGLA and TG. Compared with Cluster 1 these metabolites were 27%, 34%, 16%, 14% and 23% higher, respectively. Other probable biomarkers were identified (*p* < 0.02; *q* < 0.11), these include palmitic acid, tyrosine, β-carotene, α-carotene and betaine, which were higher in Cluster 1 by 2%, 22%, 44%, 32% and 15% (Table 2).

**Table 2.** Summary of metabolite concentration differences (average and standard deviation) between dietary clusters and performance of biomarkers. Mean concentrations (µM) for the top 15 ranked metabolites and their respective 95% confidence intervals (CI). P-values are from t-tests, q-values are false discovery rate (FDR) corrected p-values using a Bonferroni correction. Area under the receiver operating characteristic (AUROC) values indicate biomarker performance. Metabolites were deemed statistically significant if *p* < 0.05 and q < 0.05 Table 2 long description.

	Biomarker	Cluster 1 *n* = 53 (µM)	95% CI	Cluster 2 *n* = 81 (µM)	95% CI	*p*-Value	*q*-Value	AUROC	↑/↓	% change
1	*DHA	3.90 ± 1.22	± 0.33	3.03 ± 0.95	± 0.21	2.13E-5	0.001	0.72	↓	22.07
2	Adrenic acid	0.29 ± 0.64	± 0.17	0.37 ± 0.51	± 0.11	2.69E-5	0.001	0.71	↑	26.55
3	*Osbond acid	0.14 ± 0.06	± 0.02	0.19 ± 0.07	± 0.02	9.55E-5	0.003	0.69	↑	34.39
4	*EPA	2.51 ± 2.42	± 0.65	1.86 ± 2.29	± 0.50	1.74E-4	0.003	0.69	↓	25.89
5	Cholesterol	3.41 ± 0.84	± 0.23	3.95 ± 0.86	± 0.19	2.58E-4	0.004	0.68	↑	15.64
6	*DGLA	3.11 ± 0.65	± 0.17	3.55 ± 0.72	± 0.16	0.0013	0.02	0.66	↑	14.34
7	*α-linolenic acid	0.28 ± 0.11	± 0.03	0.23 ± 0.09	± 0.02	0.0023	0.03	0.65	↓	19.80
8	Citric acid	94.26 ± 33.73	± 9.08	78.05 ± 30.65	± 6.67	0.002	0.03	0.65	↓	17.19
9	Vitamin C	39.95 ± 17.58	± 4.73	31.21 ± 23.00	± 5.01	0.002	0.03	0.64	↓	21.87
10	Triglycerides	1.39 ± 0.67	± 0.18	1.72 ± 0.72	± 0.16	0.005	0.04	0.64	↑	23.12
11	Palmitic acid	31.26± 2.00	± 0.54	30.02 ± 1.62	± 0.35	0.008	0.06	0.64	↓	2.36
12	Tyrosine	5.77 ± 2.88	± 0.78	4.49 ± 2.73	± 0.59	0.009	0.07	0.63	↓	22.16
13	β-carotene	1.27 ± 1.48	± 0.40	0.71 ± 0.63	± 0.14	0.012	0.08	0.63	↓	44.05
14	α-carotene	0.46 ± 0.35	± 0.09	0.31 ± 0.20	± 0.04	0.015	0.10	0.62	↓	32.03
15	Betaine	47.83 ± 19.08	± 5.14	40.58 ± 17.77	± 3.87	0.018	0.11	0.62	↓	15.16

* Data presented as percentage of total phospholipid fraction.

Unsupervised PCA analysis incorporating all plasma metabolites (Supporting Information Figure S2.A) showed limited discrimination of Clusters 1 and 2. Principal component 1 (PC1) explained 20.5% of the variance and principal component 2 (PC2) explained 8.9%. Using the supervised method of partial least squares discriminant analysis (PLS-DA) (Supporting Information Figure S2.B) visibly improved the separation of clusters. VIP Scores revealed the top 15 most influential metabolites (Supporting Information Figure S2.C) in the PLS-DA model with 13 of the 15 tallying with the most statistically significant metabolites. PLS-DA was cross-validated using 3 components (Supporting Information Table S3).

### Biomarker performance and food-biomarker correlations

Table 2 showed the performance of individual biomarkers and Table 3 showed the best performing paired-metabolite ratios. DHA was the strongest performing individual biomarker (AUROC = 0.72) and this metabolite could be further utilised to achieve even greater biomarker performance by ratioing it with osbond acid, adrenic acid, DGLA or cholesterol (AUROC = 0.75–0.78). Table 3 showed the clear potential of leveraging DHA and osbond acid concentrations to best distinguish between Clusters 1 and 2. The paired ratio osbond acid/DHA showed the greatest AUROC (0.78) with levels differing between Clusters 1 and 2 by 72%. A graphical representation of the AUROC of DHA as an individual marker can be observed on Supporting Information Figure S3 (left) and of the AUROC of the ratio osbond acid/DHA (right).

**Table 3.** Paired metabolites ratios. Paired metabolites ratios further enhance biomarker performance. Table 3 shows the top performing paired metabolite ratios with AUROC values and % change. Clusters 1 and 2 values are mean metabolites ratio concentrations (µM) and 95% confidence intervals (CI) Table 3 long description.

	Ratio	AUROC	Cluster 1	95% CI	Cluster 2	95% CI	↑/↓	% change
1	Osbond acid / DHA	0.78	0.036	8.0E–4	0.062	6.6E–4	↑	72.22
2	Adrenic acid / DHA	0.76	0.074	6.3E–3	0.122	4.2E–3	↑	64.86
3	DGLA / DHA	0.76	0.797	0.011	1.171	0.011	↑	46.93
4	Cholesterol / DHA	0.75	0.874	0.013	1.303	0.012	↑	49.08

Table 4 showed the 15 statistically significant food-metabolite associations as determined by Spearman’s rank correlation after multiple comparisons correction using the Benjamini–Hochberg methodology. Intake of fruit, fruit juice and vegetables significantly positively correlated with DHA, EPA, α-linolenic acid, vitamin C, and betaine and negatively with adrenic acid, osbond acid, cholesterol, TG, and palmitic acid. Legume intake was significantly positively associated with alpha-carotene and negatively with DGLA. Fish intake was significantly positively correlated to DHA, EPA, vitamin C, and β and α-carotene and negatively correlated with adrenic and osbond acid, cholesterol, DGLA and TG. Red, white, and processed meat positively correlated with adrenic and osbond acid, cholesterol, and TG and negatively with citric acid and betaine.

**Table 4.** Significant food-metabolite correlations determined by Spearman’s rank correlation. Spearman’s rank correlation coefficients (r) for the top 15 most significant metabolites to indicate those significantly correlated with the individual food variables. Multiple comparison were accounted applying a Benjamini–Hochberg correction (Q). Correlations were deemed significant if *p* < 0.05 and Q < 0.05 (Benjamini–Hochberg) Table 4 long description.

Food group		Fruit, fruit juice & vegetables	Legumes	Fish	Red, white and processed meat	Whole grain cereals	Sweet foods
DHA	r	0.282	0.139	0.556	−0.179	0.197	−0.052
	p	0.001	0.111	3.70E-12	0.039	0.023	0.551
	Q	0.031	0.549	6.65E-10	0.339	0.27	0.905
Adrenic acid	r	−0.278	−0.100	−0.331	0.268	−0.191	0.061
	p	0.001	0.252	0.0001	0.002	0.027	0.485
	Q	0.031	0.680	0.006	0.056	0.285	0.867
Osbond acid	r	−0.318	0.028	−0.429	0.330	−0.121	0.086
	p	2.03 E–4	0.746	2.88E–07	1.11E–4	0.166	0.327
	Q	0.009	0.941	3.89E–5	0.006	0.623	0.746
EPA	r	0.421	−0.091	0.569	−0.133	0.201	−0.120
	p	4.60E–07	0.295	9.23E–13	0.127	0.020	0.169
	Q	4.96E–05	0.711	2.49E-10	0.586	0.257	0.623
Cholesterol	r	−0.286	0.031	−0.309	0.189	−0.112	0.031
	p	0.001	0.721	2.85E–4	0.029	0.196	0.724
	Q	0.031	0.936	0.012	0.290	0.637	0.936
Vitamin C	r	0.353	0.12	0.231	−0.201	0.332	0.187
	p	3.30E–5	0.169	0.008	0.021	9.90E-5	0.032
	Q	2.49E–10	0.623	0.160	0.257	0.006	0.308
β-carotene	r	0.117	0.138	0.305	−0.077	0.200	0.023
	p	0.178	0.111	3.38E-4	0.379	0.021	0.796
	Q	0.628	0.549	0.014	0.769	0.257	0.957
α-carotene	r	0.158	0.180	0.377	−0.044	0.153	0.005
	p	0.069	0.037	7.00 E–6	0.613	0.078	0.957
	Q	0.428	0.333	6.30E–4	0.922	0.452	0.995
Palmitic acid	r	−0.255	−0.113	−0.054	0.081	−0.096	0.178
	p	0.003	0.196	0.535	0.352	0.27	0.04
	Q	0.073	0.637	0.897	0.763	0.683	0.340
Citric acid	r	0.189	0.098	0.073	−0.147	0.175	−0.213
	p	0.029	0.258	0.404	0.090	0.044	0.013
	Q	0.290	0.680	0.802	0.490	0.349	0.206
Triglycerides	r	−0.184	−0.099	−0.251	0.217	−0.230	0.031
	p	0.033	0.255	0.003	0.012	0.008	0.722
	Q	0.312	0.680	0.073	0.202	0.160	0.936
Betaine	r	0.183	0.13	0.098	−0.020	0.078	−0.133
	p	0.035	0.135	0.26	0.814	0.155	0.126
	Q	0.746	0.934	0.905	0.972	0.290	0.767
Tyrosine	r	0.156	0.121	0.047	0.039	0.085	−0.104
	p	0.072	0.165	0.588	0.651	0.329	0.232
	Q	0.432	0.623	0.905	0.922	0.746	0.669
DGLA	r	−0.113	−0.172	−0.239	0.129	0.021	0.127
	p	0.194	0.047	0.006	0.139	0.807	0.146
	Q	0.637	0.357	0.135	0.607	0.958	0.611
α-linolenic acid	r	0.181	0.01	0.037	−0.201	0.195	−0.149
	p	0.037	0.911	0.675	0.021	0.025	0.087
	Q	0.333	0.981	0.922	0.257	0.281	0.479

## Discussion

The present investigation demonstrates the effectiveness of K-means cluster analysis to derive dietary patterns from a study population using food diary data, and how concomitant blood samples can be used to uncover biomarkers reflecting adherence to the resulting dietary patterns. To the best of our knowledge, this is the first time that K-means cluster analysis has been applied in this way.

Participants included in the present analysis were engaged in an ongoing dietary intervention (MEDDINI study) during sample collection, reflecting the longitudinal nature of the original trial. However, for this analysis, biomarker data were evaluated cross-sectionally without consideration of specific timepoints.

Our search for potential blood biomarkers of healthy eating involved a dual strategy. First, a pre-defined list of 31 commonly measured food-derived nutrients and diet-related CVD risk factors were tested. Each of these have previously been investigated as possible dietary biomarkers, and specifically within our research group as indicators of compliance to a Mediterranean dietary pattern.^(27)^ Second, a non-targeted 1D 1H-NMR metabolomic approach identified and quantified 59 metabolites. Our investigations also tested all 540 potential food-metabolite correlations to detect relationships between individual blood metabolites and dietary patterns. Only 17 significant associations emerged following multiple comparisons correction, of which 15 corresponded to the top-most significant listed metabolites (Table 4). These data offer wider insights about biomarker utility within the context of a complex diet.

The objectivity of quantitative methods is a key advantage for measuring human dietary patterns, and K-means clustering is a robust^(28)^ and reproducible method^(29)^ widely used to derive dietary patterns. It aims to create homogenous groups by maximising the Euclidian distance between the cluster centres and minimising the distance between the individuals around the centre of their closest cluster.^(30)^ Subjectivities, such as the selection of the cluster numbers or which data to include, can be overcome by stepwise optimisation and rigorous cross-validation as described here. Food intake data are usually inputted to clustering analysis in one of three ways: (i) the frequency of the food consumed (servings) (ii) the percentage total energy contribution from food (%TE Food), or (iii) the portion size of the food consumed (grams).^(11)^ The present study opted to input food data in grams as food portions and are described in detail in the MEDDINI food diaries, with all amounts databased in grams, which could be easily standardised prior to clustering. The clustering analysis derived two distinct dietary clusters or patterns with 6 food groups. Cluster 1 might be described as a ‘healthy dietary pattern’. Indeed, a very similar dietary pattern (high intake of fish, whole grains; low intake of refined products, sugar and sweets and cured meat) was associated with a lower risk of metabolic syndrome and lower risk of low HDL cholesterol in normal weight individuals.^(31)^ Conversely, Cluster 2 could be described as ‘Westernised’ because it is perceivably lower in fibre (i.e. 50% less fruit and vegetables and 38% less whole grain cereals), higher in refined sugar (i.e. 123% more sweet foods) and higher in saturated fat (i.e. 63% more meat and 66% less fish).

These results supported the validity of the 2-cluster, 6-food-group model, which consistently produced stable clustering patterns and met convergence criteria in at least 8 out of 10 validations. Models with fewer food groups failed to form meaningful clusters, while those with more than 6 led to unbalanced sample sizes – highlighting the need for a clustering solution that was statistically robust and dietary-pattern representative. This aligns well with previous research noting that there is no universal standard for determining cluster number, necessitating researchers to define meaningful clusters of reasonable sample sizes.^(11)^

The approach undertaken builds on that of O’Sullivan et al.^(24)^ who applied five-fold cross-validation by removing one-fifth of the data per fold. In contrast, we used a stricter three-fold method, randomly excluding one-third of the data and repeating the process 10 times. Despite the small sample size and dietary variability, this tested the stability of the clustering framework and confirmed consistent results across data subsets.

No significant differences were observed in age, BMI, smoking status, sex distribution, or systolic blood pressure. Although a statistically significant difference was found in diastolic blood pressure (*p* = 0.014), the absolute difference (5.27 mmHg) remained within normal limits and was unlikely to influence clinical outcomes.

Graphical representations – including a mean plot with 95% CIs, and PCA centroid plots – highlighted distinct dietary behaviours between clusters. Despite the noted variability, the absence of overlap in CIs and the significant differences confirmed by t-tests (*p* < 0.05) across all food groups underscored the distinct nature of the clusters which reflected distinct dietary habits or preferences between the clusters. The PCA plot showed distinctly separated groupings, and introducing a third cluster did not enhance separation, supporting the choice of the 2-cluster model.

Statistical comparisons found plasma levels of 10 metabolites to significantly differ between the two clusters, and five further metabolites were also identified differing between the 2 clusters. DHA was the most statistically significant, the most influential in discriminating clusters in multivariate analysis (as determined by PLS-DA VIP scores) and was the strongest performing individual biomarker (as determined by AUROC).

DHA was selected as a reference metabolite due to its strong discriminative performance (AUROC = 0.72, Table 2) and its established association with healthy dietary patterns adherence.^(17)^ Its use as a denominator enhanced classification power when paired with other metabolites, particularly Osbond acid (AUROC = 0.78), and improved the distinction between dietary patterns. DHA is a well-known fatty acid found in fish and has previously been reported as a marker of fish intake.^(32,33)^ Our findings supported this, showing a strong positive correlation with fish intake (r = 0.556; *p* = 6.7E–10), among the highest observed. DHA also significantly correlated with fruit and vegetable intake, likely due to frequent co-consumption. In biomarker research, such associations may not reflect direct intake. EPA showed similar patterns, correlating with the same food types – fish, and fruit, fruit juice and vegetables. This is interesting because one Chinese study has noted that combined EPA and DHA levels associated closely to a ‘fish–vegetable’ dietary pattern score,^(34)^ and another Nordic study correlated DHA with fish and fruit intake, and EPA with fish (but not fruit) intake.^(17)^

The omega-6 fatty acid osbond acid (ω−6 docosapentaenoic acid) has been proposed to enhance DHA biomarker performance.^(35)^ Omega-6 fatty acids are primarily in red meat, poultry, and eggs,^(36)^ and the presence of osbond acid has been reported in meat.^(37)^ Osbond acid levels in erythrocyte membranes correlated positively with meat intake and negatively with fish intake,^(38)^ which was consistent with our findings here. Osbond acid was also significantly elevated in Cluster 2 (*p* = 9.55E–5), which had higher meat intake. Similarly, adrenic acid (docosatetraenoic acid) showed significant higher levels in Cluster 2 (*p* = 2.69E–5). Although it was significantly correlated to meat intake it lost significance when multiple comparisons corrections were applied. Adrenic acid is present in red and white meat^(39,40)^ and in processed meat,^(41)^ and has been linked to NAFLD progression in both clinical and animal studies.^(42)^

The samples and data used in the present study were obtained from the MEDDINI intervention, which promoted adherence to a Mediterranean dietary pattern. Fruit and vegetable intake, a central component of this pattern, was a key discriminating factor in the K-means cluster analysis. Cluster 1 included participants whose fruit, fruit juice, and vegetable intake was over 50% higher than in Cluster 2. Among the shortlisted biomarkers, few strongly correlated with this intake. Vitamin C correlated positively with both fruit/vegetable and wholegrain cereal intake, likely reflecting adherence to a healthy dietary pattern. Citric acid and betaine also associated with fruit and vegetable intake, which was consistent with previous studies,^(43–46)^ though significance was lost after multiple comparison correction. Still, our findings aligned with those of Gibbons et al., who also found citric acid and betaine significantly associated with a healthy dietary pattern using cluster analysis in 567 individuals. Their clusters were similar to ours: a ‘healthy’ cluster high in fruit, vegetables, fish, and whole grains, and an ‘unhealthy’ cluster rich in processed foods and sugary drinks. As in our study, citric acid and betaine levels were higher in the healthy cluster.^(16)^ Alpha- and β-carotene, though previously linked to fruit/vegetable intake,^(7)^ did not show strong associations here, but instead unexpectedly correlated with fish. Notably, the most consumed fruits were apple, pear, and banana which possess a lower carotenoid content;^(47,48)^ hence the heterogeneity of fruit and vegetables should be taken into consideration. Aggregating foods into broader categories, as done here, may also affect biomarker associations. While carotenes occur in fish, their concentrations are much lower than in fruits and vegetables.^(49)^ Carotenoids also differ in their associations: lutein is linked to vegetable intake while beta-cryptoxanthin to fruit.^(50,51)^ Further research is needed to identify robust biomarkers of fruit and vegetable intake. Vitamin C is not universally present in these foods, and a biomarker panel may be preferable.^(7,52)^ Our previous work found citric acid to be the most influential metabolite distinguishing adherence to a MD,^(21)^ and it was reassuring to see that it was a moderately good biomarker for dietary Cluster 1.

The omega-6 fatty acid DGLA was associated with the least healthy dietary pattern (Cluster 2), showing significant negative correlations with legumes and fish intake. Conversely, α-linolenic acid (omega-3) was higher in Cluster 1 and positively correlated with fruit, vegetables, and whole grains, and negatively with meat intake, though these associations lost significance after correction. Major sources of α-linolenic acid include dairy, fruits, vegetables, fats, oils, and cereals.^(53–56)^ While fats and oils were not quantified here, higher cereal intake in Cluster 1 supports these findings. DGLA, found in red and white meat,^(41,57)^ did not show a significant direct association with meat intake. Its metabolic role remains unclear, with reports linking it to both anti-inflammatory effects^(58)^ and negative outcomes such as cognitive decline,^(59,60)^ insulin resistance,^(61)^ and type 2 diabetes.^(62)^ A meta-analysis of 3595 participants found triglycerides (TG) were significantly reduced by fish and nut intake, with the highest SUCRA values (97% and 78%), followed by red meat (72%), legumes (58%), and whole grains (53%). In our study, TG were significantly higher in Cluster 2, which had greater intake of meat and sweet foods, and lower intake of fish, fruits, and legumes. TG levels correlated negatively with fish intake, in line with the meta-analysis.^(63)^ While red meat was grouped with white and processed meat here, TG showed a positive correlation with this combined category.

In the current study, the examined biomarkers were selected by a pre-defined list of food-derived nutrients and diet-related CVD risk factors and a list of metabolites identified by a non-targeted 1D 1H-NMR metabolomic approach and both sets of data were obtained from the MEDDINI MD intervention study. K-means proved to be a useful approach in deriving distinct clusters and assigning individuals to each of the derived clusters, however, the present study had several limitations. The study was inherently limited by the fixed number of available metabolites. However, this dataset reflected the full extent of measurable biomarkers derived from all nutritional markers and CVD risk factors measured in the original MEDDINI study and all recoverable metabolites from non-targeted ^1^H-NMR profiling ensuring the inclusion of available biomarkers. Although analyses were controlled for potential biomarker-confounding factors, causality cannot be inferred from the results due to the cross-sectional nature of the study, therefore further research should be carried out to observe whether the proposed biomarkers reflect long-term intake. Food items were grouped based on the MEDDINI study’s dietary advice. Vegetables, fruits, and fruit juices were combined based on the recommendation to increase their intake. This is well justified given the typical co-consumption of these foods. Conversely, due to the advised reduction in red and processed meat consumption and the limited intake of poultry to two servings per week, these protein sources were combined into a single group. While the present study maximised the inclusion of food groups to derive the most sensible dietary patterns by grouping dietary variables, 3 food groups were excluded from the analysis.

Nuts were excluded from the analysis as they were consumed by only 7.5% of participants, with an average intake of 0.73 g/day, significantly lower than the 30 g/day typically associated with health benefits.^(64,65)^ Olive/rapeseed oil and spreads, although consumed by 64.2% of participants, had an average intake of only 2.33 g/day, which is markedly lower than the 20–50 g/day reported in dietary interventions.^(66,67)^ Alcohol was consumed by 47% of participants, with a highly skewed distribution (skewness coefficient = 2.64). The average of 89.02 g/day was heavily influenced by extreme values, further justifying its exclusion to prevent distortion of clustering outcomes.

When utilising repeated samples from individual participants there is potential for statistical bias to be introduced. A detailed breakdown of sample distribution across the timepoints can be found in the Supporting Information Table S2. To mitigate this bias, we employed a 3-fold cross-validation which confirmed that the clusters were stable. Reassuringly, the clustering results reflected the dietary changes observed in the original MEDDINI intervention. Cluster 1 (termed as ‘healthy’) mainly included plasma samples from the 6- and 12-month timepoints, after diet improvements had occurred. Cluster 2 (termed as ‘unhealthy’) mostly included baseline samples before the intervention had taken place. Such a distribution followed the intervention’s timeline, with participants improving their adherence from baseline to 6 months and maintained it up to 12 months. This pattern further supports the clustering approach undertaken here. The moderate performance metrics reported in Supporting Information Table S3 showed that the analytical approach undertaken had limitations. While increasing *R* ^2^ values with additional components suggested greater model complexity, the concomitant decline in *Q* ^2^ values indicated potential overfitting and reduced predictive ability. The absence of CIs (due to software limitations) made it difficult to assess overall variability and model stability. The PCA plot (Figure S2A) showed low discriminative ability, whereas PLS-DA (Figure S2.B) achieved clearer separation of the clusters. The VIP scores (Figure S2.C) identified the most influential metabolites contributing to this separation, which aligned with the top-most significant biomarkers identified by the univariate analysis findings.

In conclusion, this study identified at least 10 (and up to 15) blood metabolites potentially useful for assessing dietary patterns. Higher levels of DHA, EPA, α-linolenic acid, citric acid, vitamin C, palmitic acid, tyrosine, β-carotene, α-carotene, and betaine, and lower levels of adrenic acid, osbond acid, cholesterol, DGLA, and TG appear indicative of a healthier diet. The alignment of our results with previous metabolomic studies supports their potential as a biomarker panel in nutrition research. Future steps include validating these biomarkers and ratios in larger cohorts and testing their responsiveness to controlled dietary changes. Once confirmed, they could be added to dietary biomarker libraries and used in nutritional assessment and dietary classification.

## Supporting information

10.1017/jns.2026.10115.sm001 Macias et al. supplementary material Macias et al. supplementary material

## Supplementary material

The supplementary material for this article can be found at https://doi.org/10.1017/jns.2026.10115.

## Figure 1. Long description

The bar graph compares average consumed food in grams per day across two clusters, with six food types represented by different colors. The x-axis labels the two clusters, while the y-axis shows the average consumed food in z-scores. The food types include fruit, fruit juice and vegetables, legumes, fish, red, white and processed meat, cereals, and sweets. Cluster 1 shows higher consumption of fruit, fruit juice and vegetables, legumes, and fish, while Cluster 2 shows higher consumption of red, white and processed meat, cereals, and sweets. All values are approximated.

Navigate back to Figure 1..

## Figure 2. Long description

A two line graph compares mean food group consumption by cluster with 95% confidence intervals. The x-axis represents different food groups: fruit, fruit juice, and vegetables; legumes; fish; red, white, and processed meat; cereals; and sweets. The y-axis measures mean consumption in grams per day. Cluster 1 is represented by blue dots, and Cluster 2 by green squares. Cluster 1 shows significantly higher consumption of fruit, fruit juice, and vegetables, with a mean around 600 grams per day. Cluster 2 has higher consumption of legumes, fish, red, white, and processed meat, and cereals, with means ranging from approximately 50 to 150 grams per day. The error bars indicate 95% confidence intervals, with non-overlapping intervals highlighting distinct dietary patterns between the clusters. All values are approximated.

Navigate back to Figure 2..

## Table 1. Long description

The table presents a comparison of average daily food consumption between two clusters, labeled Cluster 1 and Cluster 2. It includes six food types: fruit, fruit juice and vegetables; legumes; fish; red, white and processed meat; wholegrain cereals; and sweet foods. Each food type is listed in a row, with columns for the average consumption in grams per day for each cluster, their respective 95% confidence intervals, and p-values indicating statistical significance. Cluster 1 has 53 individuals, while Cluster 2 has 81. Notable trends include significantly higher consumption of fruit, fruit juice, and vegetables in Cluster 1 compared to Cluster 2, with an average of 569.44 grams per day versus 281.30 grams per day. Legumes and fish are also consumed more in Cluster 1. In contrast, Cluster 2 shows higher consumption of red, white and processed meat, wholegrain cereals, and sweet foods. The p-values indicate significant differences in all food variables between the two clusters, with the most notable being the consumption of fruit, fruit juice, and vegetables, and legumes.

Navigate back to Table 1..

## Table 2. Long description

The table presents a comparison of metabolite concentrations between two dietary clusters, highlighting average values, standard deviations, and 95% confidence intervals. It includes 15 metabolites ranked by their significance, with p-values and q-values indicating statistical significance. The area under the receiver operating characteristic (AUROC) values assess biomarker performance. Notable trends include higher levels of DHA, EPA, linolenic acid, citric acid, and vitamin C in Cluster 1, while Cluster 2 shows higher levels of adrenic acid, osbond acid, cholesterol, DGLA, and triglycerides. Other biomarkers like palmitic acid, tyrosine, beta-carotene, alpha-carotene, and betaine are also identified with their respective percentage changes.

Navigate back to Table 2..

## Table 3. Long description

The table presents a comparison of the performance of various paired metabolite ratios. It includes columns for the ratio names, AUROC values, cluster concentrations with 95% confidence intervals, direction of change, and percentage change. The ratios listed are Osbond acid / DHA, Adrenic acid / DHA, DGLA / DHA, and Cholesterol / DHA. Osbond acid / DHA has the highest AUROC value of 0.78, with a 72.22 percentage change and cluster concentrations of 0.036 and 0.062. Adrenic acid / DHA follows with an AUROC of 0.76, a 64.86 percentage change, and cluster concentrations of 0.074 and 0.122. DGLA / DHA has an AUROC of 0.76, a 46.93 percentage change, and cluster concentrations of 0.797 and 1.171. Cholesterol / DHA has an AUROC of 0.75, a 49.08 percentage change, and cluster concentrations of 0.874 and 1.303. All ratios show an increase in the direction of change.

Navigate back to Table 3..

## Table 4. Long description

The table presents significant food-metabolite correlations determined by Spearman's rank correlation. It includes six food groups: fruit, fruit juice and vegetables, legumes, fish, red, white and processed meat, whole grain cereals, and sweet foods. The table has 15 rows for different metabolites and six columns for the food groups, with correlation coefficients (r), p-values, and Q-values for each metabolite-food group pair. Notable trends include positive correlations of fruit, fruit juice, and vegetables with DHA, EPA, alpha-linolenic acid, vitamin C, and betaine, and negative correlations with adrenic acid, osbond acid, cholesterol, triglycerides, and palmitic acid. Legume intake is positively associated with alpha-carotene and negatively with DGLA. Fish intake shows positive correlations with DHA, EPA, vitamin C, and beta-carotene, and negative correlations with adrenic and osbond acid, cholesterol, DGLA, and triglycerides. Red, white, and processed meat intake is positively correlated with adrenic and osbond acid, cholesterol, and triglycerides, and negatively with citric acid and betaine.

Navigate back to Table 4..
