# Supplementary material for: Identification of blood biomarkers of a healthy dietary pattern as facilitated by cluster analysis in patients from the MEDDINI study: a pilot randomised trial
Source: J Nutr Sci. 2026 Jul 7;15:e52. doi: 10.1017/jns.2026.10115 (PMC13369256; doi:10.1017/jns.2026.10115)
Supplement: Macias et al. supplementary material [file S2048679026101153sup001.docx]

SUPPORTING INFORMATION

Table S1.A. Baseline Characteristics of the study participants from the MEDDINI study for the present study n=57

| **Variable** | **Baseline participants (n=57)** |
| --- | --- |
| **Age (years) Mean (SD)** | 57.40 (9.10) |
| **BMI (Kg/m2) Mean (SD)** | 30.28 (5.36) |
| **BP. Systolic (mmHG) Mean (SD)** | 124.34 (17.61) |
| **BP. Diastolic (mmHG) Mean (SD)** | 68.01 (9.66) |
| **Smokers %** | 20.00 |
| **Sex: Male %** | 80.00 |
| **Sex: Female %** | 20.00 |

Summary of the baseline characteristics of participants from the original MEDDINI intervention study used in the present study (n=57). Mean age was 57.40. Mean BMI was 30.28 (participants were in the range of overweight and Class I obesity at baseline). Systolic and Diastolic blood pressure were 124.34 and 68.01 mmHg respectively. Twenty percent of participants were smokers, 80% were male and 20% female.

Table S1.B. General characteristics of participants included in Cluster 1 (n=53) and Cluster 2 (n=81).

| **Variable** | **Cluster 1 n=53** | **Cluster 2 n=81** | **p-value** |
| --- | --- | --- | --- |
| **Age (years) Mean (SD)** | 57 (8.14) | 56.68 (9.00) | 0.93 |
| **BMI (Kg/m2) Mean (SD)** | 30.25 (6.56) | 30.07 (4.73) | 0.85 |
| **BP. Systolic (mm HG) Mean (SD)** | 128.07 (20.03) | 123.83 (19.27) | 0.22 |
| **BP. Diastolic (mm HG) Mean (SD)** | 75.23 **(**12.34) | 69.96 (10.94) | *0.014 |
| **Smokers %** | 11.32 | 16.05 |  |
| **Sex: Male %** | 84.91 | 76.54 |  |
| **Sex: Female %** | 15.09 | 23.46 |  |

Summary of the characteristics of participants in Cluster 1 and Cluster 2. Mean age was 57 for the Cluster 1 and 56.68 for the Cluster 2. Mean BMI was 30.25 for Cluster 1 and 30.07 for Cluster 2. No significant differences were found for age, BMI, smoking status, sex distribution, and systolic blood pressure between clusters (p>0.05).

Specifically, between the two clusters, 10 participants had samples from 1 timepoint, 17 participants had samples from 2 timepoints, and 30 participants had samples from 3 timepoints. This distribution is as follows:

- 10 participants × 1 timepoint = 10 unique samples (10 participants represented once)
- 17 participants × 2 timepoints = 34 samples (17 participants represented twice)
- 30 participants × 3 timepoints = 90 samples (30 participants represented three times)
- **Total = 134 samples from 57 unique participants**

**Table S2. Distribution of Participants and Samples in Each Cluster**

| **Cluster** | **Unique Participants** | **Participants with 1 Sample** | **Participants with**  **2 Samples** | **Participants with**  **3 Samples** | **Total Samples** |
| --- | --- | --- | --- | --- | --- |
| **Cluster 1**  **"Healthy"** | 34 | 1 | 11 | 22 | 53 |
| **Cluster 2**  **"Unhealthy”** | 50 | 9 | 15 | 26 | 81 |
| **Total unique participants** | 57 | 10 | 17 | 30 | 134 |

Table S3. Performance of multivariate modelling of metabolomic data.

| Measure | 1 component | 2 components | 3 components |
| --- | --- | --- | --- |
| Accuracy | 0.652 | 0.634 | 0.620 |
| R2 | 0.271 | 0.351 | 0.516 |
| Q2 | 0.095 | 0.091 | 0.054 |

Cross validation of the PLS-DA multivariate model as performed using Metaboanalyst.


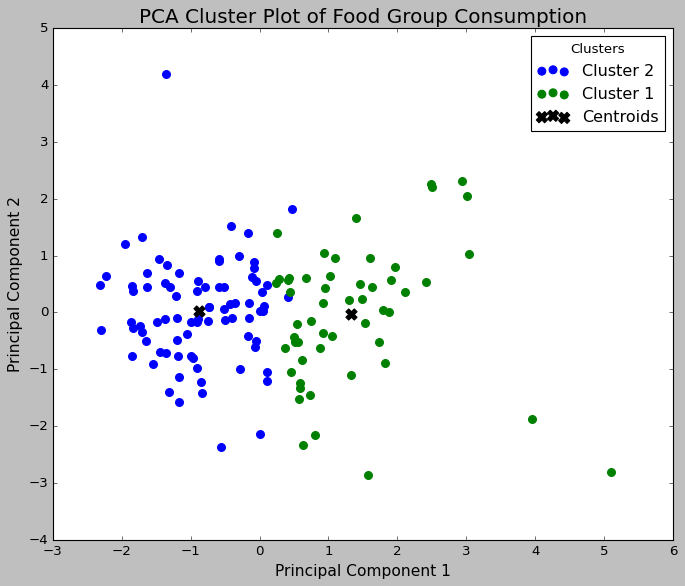

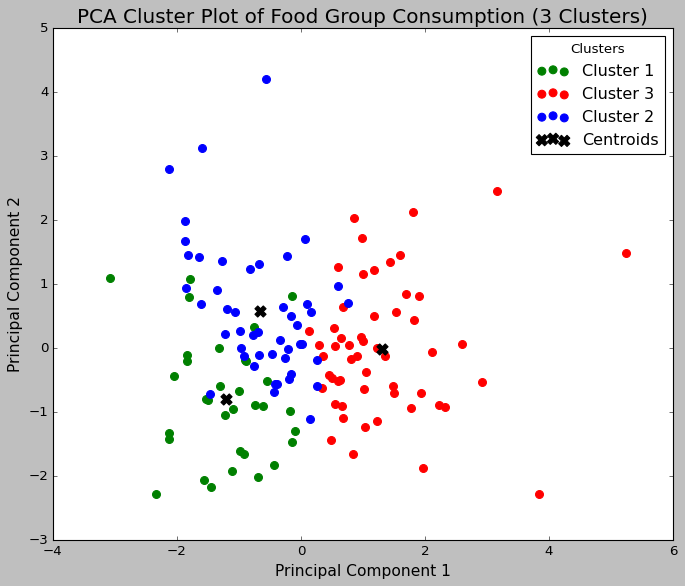


Figure S1: PCA Scatter Plots of Food Group Consumption for Two and Three Cluster Solutions with Centroids. The left plot displays participants in two-dimensional PCA space for the two-cluster model, with blue and green points representing Clusters 1 and 2, respectively. The plot on the right illustrates the same PCA space applied to the three-cluster model, with the addition of red points for Cluster 3. Black stars indicate the centroids, summarizing the central location of each cluster. Both plots show the dietary pattern distinctions captured by the k-means clustering within our study population.

| **A** | **B** | **C** |
| --- | --- | --- |
| 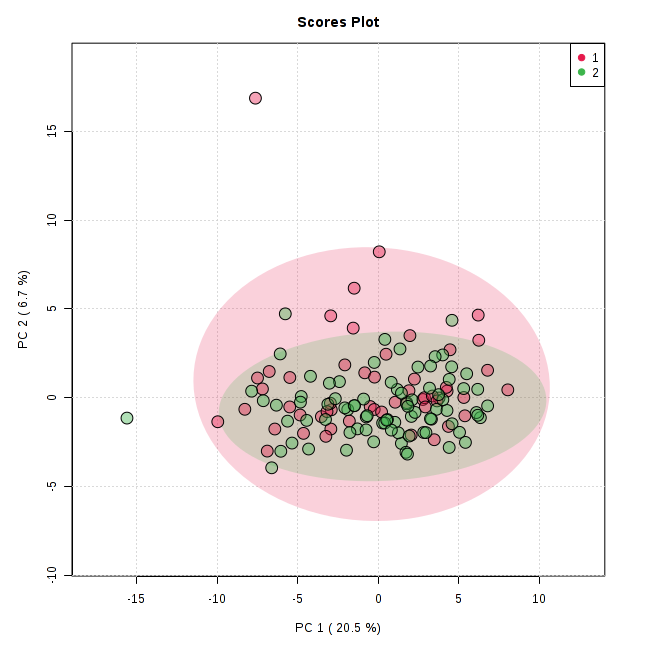 | 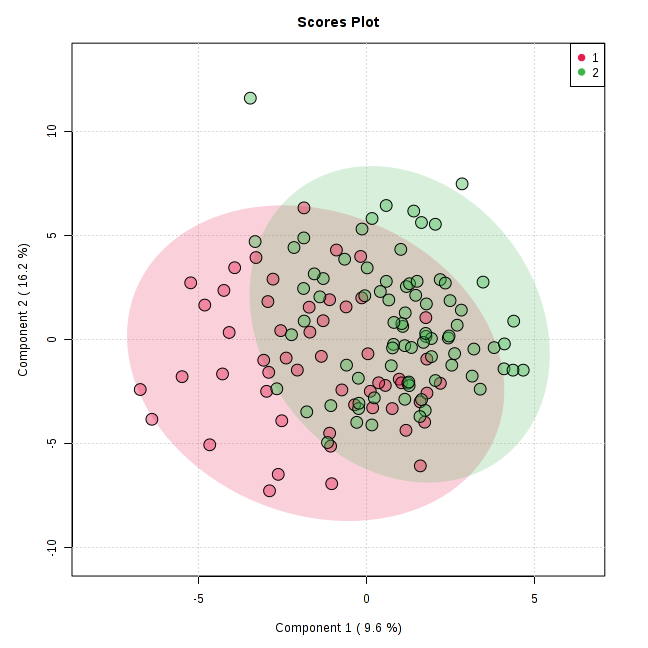 | 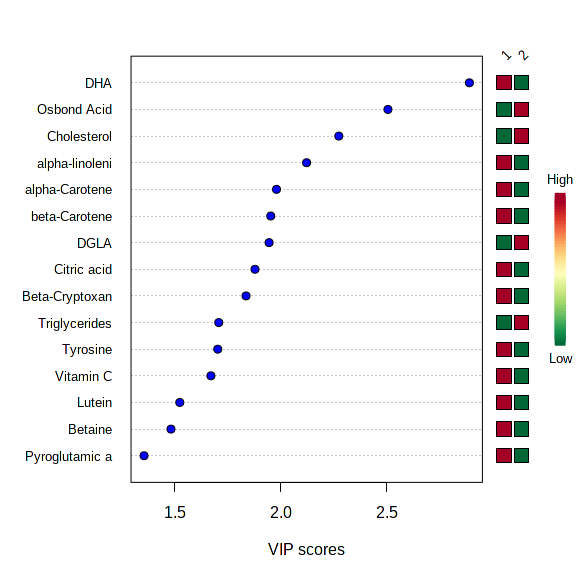 |

Figure S2. Multivariate statistical modelling of plasma metabolite data incorporating 134 samples each with 90 metabolite measurements. Panel A shows separation of dietary clusters by principal component analysis (PCA) and panel B by partial least squares discriminant analysis PLS-DA. Cluster 1 samples are represented in red. Cluster 2 samples are indicated in green with 95% confidence intervals indicated. Panel C shows the variable importance in projection (VIP) scores for the 15 most influential metabolites for separations observed in the PLS-DA model. Analysis was performed using Metaboanalyst version 4 and metabolite concentration data were auto-scaled prior to analysis.


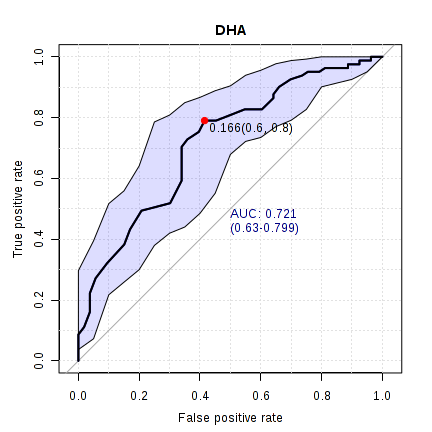

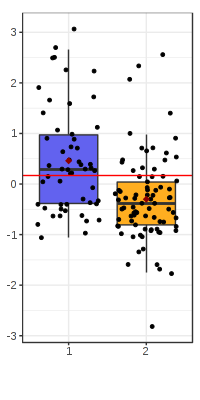

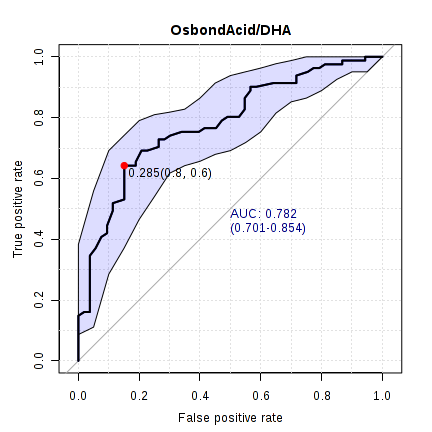

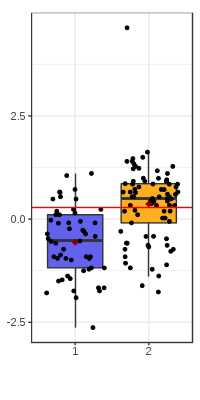


Figure S3. DHA biomarker performance and ratio DHA/Osbond acid area under the ROC curve. DHA showed the strongest biomarker performance for a healthier dietary pattern and its performance was enhanced the most by ratioing with osbond acid. Metabolite data median-centred and analysed using Metaboanalyst (version 4). As a single biomarker DHA achieved the highest AUCROC (0.72) with levels differing between Clusters 1 and 2 by 22%. However, paired ratio of this metabolite with osbond acid (osbond acid/DHA) showed the greatest AUCROC (0.78) with levels differing between Clusters 1 and 2 by 72%. Optimal cut-off is represented on ROC curves by a red dot and by a red horizontal line on box plots. The Y-axis of box plots represents concentrations in µM and mean values for each group are indicated by a red diamond.
